# Negative Pressures and the First Water Siphon Taller than 10.33 Meters

**DOI:** 10.1371/journal.pone.0153055

**Published:** 2016-04-07

**Authors:** Francisco Vera, Rodrigo Rivera, Diego Romero-Maltrana, Jaime Villanueva

**Affiliations:** Instituto de Física, Pontificia Universidad Católica de Valparaíso, Valparaíso, Chile; University of Southern California, UNITED STATES

## Abstract

A siphon is a device that is used to drain a container, with water rising inside a hose in the form of an inverted U and then going down towards a discharge point placed below the initial water level. The siphon is the first of a number of inventions of the ancients documented about 2.000 years ago by Hero of Alexandria in his treatise Pneumatics, and although the explanation given by Hero was essentially correct, there is nowadays a controversy about the underlying mechanism that explains the working of this device. Discussions concerning the physics of a siphon usually refer to concepts like absolute negative pressures, the strength of liquid’s cohesion and the possibility of a siphon working in vacuum or in the presence of bubbles. Torricelli understood the working principle of the barometer and the impossibility of pumping water out of wells deeper than 10.33 m. Following Torricelli’s ideas it would also not be possible to build a siphon that drives pure water to ascend higher than 10.33 m. In this work, we report the first siphon that drives water (with surfactant) to ascend higher than the Torricellian limit. Motivated by the rising of sap in trees, we built a 15.4 m siphon that shows that absolute negative pressures are not prohibited, that cohesion plays an important role in transmitting forces through a fluid, and that surfactants can help to the transport of water in a metastable regime of negative pressures.

## Introduction

Intrigued about the existence of the vacuum, in 1644 Torricelli invented the mercury barometer, establishing a conceptual connection between the weight of our atmosphere and the impossibility of pumping water out of wells deeper than 10 m. Torricelli realized that the production of a partial vacuum at the top of the extracting tube allowed the atmospheric pressure at the lower part of the well to push the liquid to rise towards the lower pressure zone produced by the pump. Thus, it would be impossible to pump or to support a water column higher than 10.33 m inside a siphon or a barometer, since this would imply absolute negative pressures. Nevertheless, mercury barometers with absolute negative pressure exist [1]. Moreover, about one hundred years ago the currently accepted explanation for the rising of sap inside trees challenged Torricelli’s ideas [2]. Nowadays, it is well established that tiny pores in the leaves can produce negative pressures inside the xylem of a tree which can pull water columns towards heights much larger than the Torricellian limit.

Capillary forces produced at the pores in the leaves are transmitted by cohesive forces between water molecules and can sustain the weight of water columns greater than one hundred meters. Water inside the capillaries in the xylem is pumped due to transpiration at the leaves in a process described by the cohesion tension theory [3–5]. Water under absolute negative pressures should boil at ambient temperature forming bubbles that would eliminate the cohesive forces. However, nature provides trees with some not completely well understood mechanisms that forbid bubble nucleation, and that even make possible to refill the capillaries after an embolism [6, 7].

The effects of forces produced by pressure differences on fluids are typically explained using only pushing forces because many people do not believe that fluids are capable of pulling. Some years ago a report in Discovery Magazine about the mechanism used by trees to raise water mentioned the existence of negative pressures and produced criticisms of several scientists against the journalist. Fortunately, she wrote a second column [8] after asking the opinion of respected scientists in the fields of physics and botany, resulting in a story full of contradictions between experts. The main conclusion was that negative pressures do exist, and that the physics community still has problems in accepting the existence of negative pressures that are a well established fact for botanists.

The idea of a fluid that can only push follows naturally from the “ideal gas model”, where the only mechanism available for momentum transfer is by means of collisions between atoms or molecules. In this scenario it is possible to diminish the pressure by lowering the density of the fluid, up to the point where there is no longer any fluid left, or by lowering the kinetic energy of its molecules, up to the point where all molecules stop and there is no exchange of momentum. In this model both cases correspond to a situation of zero pressure, which cannot be further lowered. It is in this context where pulling forces or negative pressures are absurd. However, if there are attractive forces pulling (instead of pushing) molecules or atoms, then negative pressures are perfectly well defined. Because attractive intermolecular forces do exist, negative pressures are expected to appear when liquids are pulled. It has to be noted that, although the topic is controversial, negative pressures are frequently measured to catalog properties of liquids [4, 9–12].

The underlying conceptual connection between the pumping of sap in trees and the explanation given by Hero on the working mechanism behind siphons, provided the motivation to try to demonstrate the existence of absolute negative pressures by building the first siphon capable of raising water over the supposedly impossible height of 10.33 m.

## The driving force in siphons

Hero correctly explained that siphons are driven by a weight imbalance between water columns [13], but nowadays there is a debate between people who believe that siphons are driven by pressure differences and people who believe that siphons are driven by the force of gravity. The discussion is also polarized between people who believe that the pushing mechanism of pressure is the only mechanism transmitting forces inside fluids, and those who believe that molecular pulling forces can also play a relevant role [14–19].

**Fig 1:**
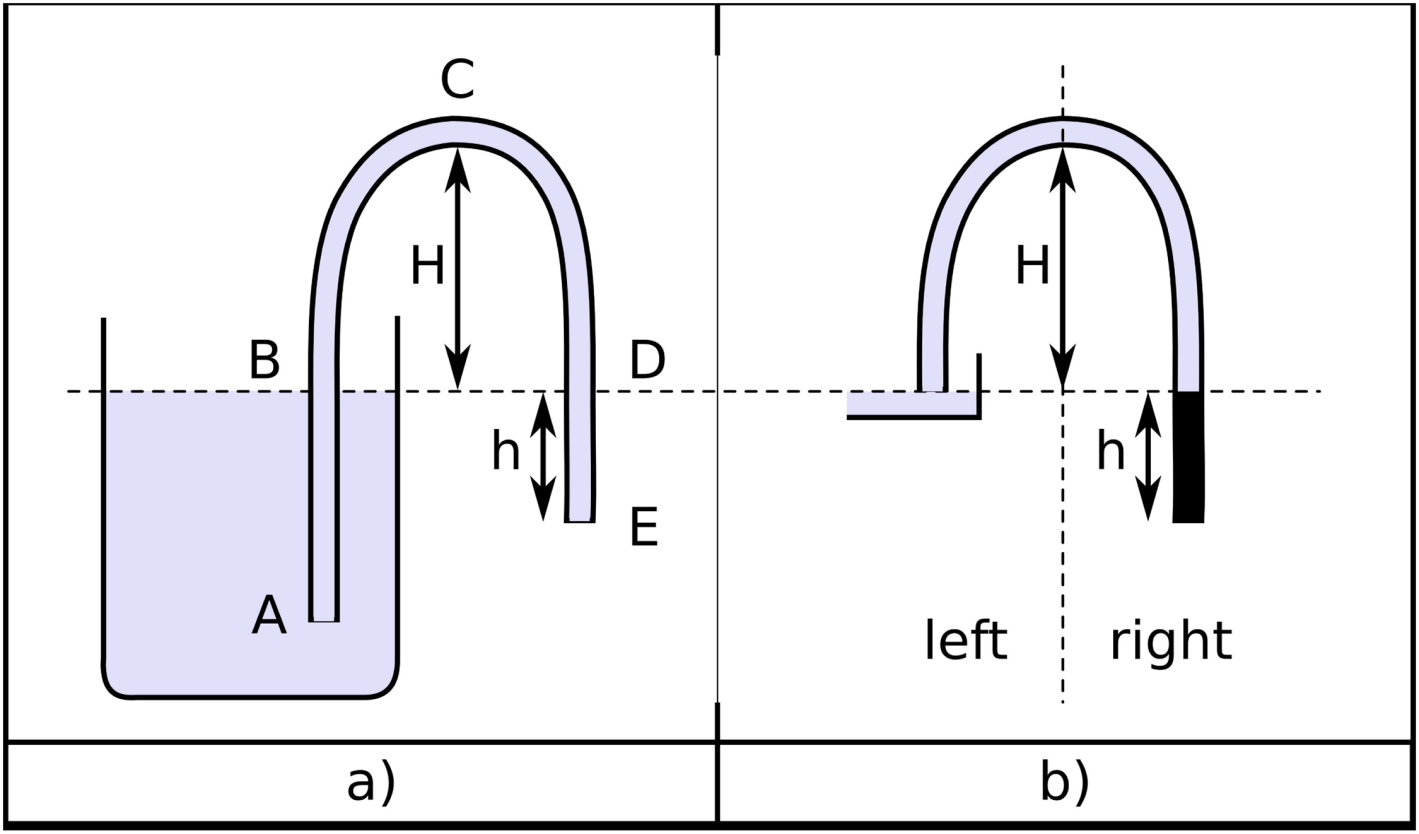
a) A simple siphon. b) Minimalist version of the siphon shown in (a) that highlights the driving mechanism of siphons.

To identify the driving force that moves water in a siphon, we will start by considering the static case where the discharging end of the hose is maintained closed using a stopper. In this situation the stopper provides a force that makes it possible to produce zero net forces on any portion of the liquid. In this static situation the pressure inside the hose at points B and D in Fig 1 corresponds to the atmospheric pressure *p*_0_, and the weight of the liquid inside the segment of the hose between D and the discharging end of the hose at E (shown in black in the minimalist siphon of Fig 1b) plus the force on this segment due to the atmospheric pressure at D are exactly balanced by the force produced by the stopper at E. After removing the stopper, the weight of this portion of liquid (that corresponds to the net force on this segment) causes it to fall, which in turn diminishes the pressure at D producing a pressure gradient that drives the siphon. Thus, the value of the atmospheric pressure *p*_0_ is irrelevant and the force that maintains the fluid in motion can emerge from pushing forces produced by collisions of air molecules at the open surface of the fluid at B and/or from the pulling produced by cohesive forces between the liquid’s molecules that are transmitted from the falling portion of the fluid.

Regions of absolute negative pressures are present in mercury barometers with columns taller than 760 mm and in mercury siphons working in a vacuum [9, 20]. Recently a siphon using an ionic liquid was demonstrated to transfer liquid from one container to another inside a vacuum chamber [21]. This siphon provides an excellent example that shows that the atmospheric pressure is irrelevant, because in this experiment there are no external pushing forces and the underlying force that drives the fluid to move can only be the gravitational force. Besides, the initial motion of the unbalanced portion of fluid can only be transmitted by cohesive pulling forces. On the other hand, searching the web for “Pouring and Siphoning a Gas” it is possible to find a video from Flinn Scientific of a gas siphon that provides an excellent example of siphoning action transmitted solely by pushing forces related to atmospheric pressure, in which the initial pressure gradient –and the source of energy keeping the fluid in motion– is produced again by the weight of the unbalanced portion of gas shown schematically in the right section of Fig 1b. All siphons are driven by the weight of this unbalanced column and, taking aside extreme examples of siphons in vacuum or gas siphons, the forces transmitted inside the fluid are a mix of pressure forces that push and cohesive forces that pull.

## Bubbles and our first experiments

It is common for trees to suffer and to recover from embolisms, but for siphons bubble formation in a regime of negative pressures is a lethal problem. Bubbles can exist in a siphon working in regimes of positive pressures as long as the weights of the fluid inside the right and left sections of the hose are different. A siphon raising water higher than 10.33 m should have regions of negative pressures where the fluid is in a metastable state and can boil at ambient temperature. For these siphons, the presence or the nucleation of a bubble would disconnect the cohesive forces and therefore the siphoning action would stop.

**Fig 2:**
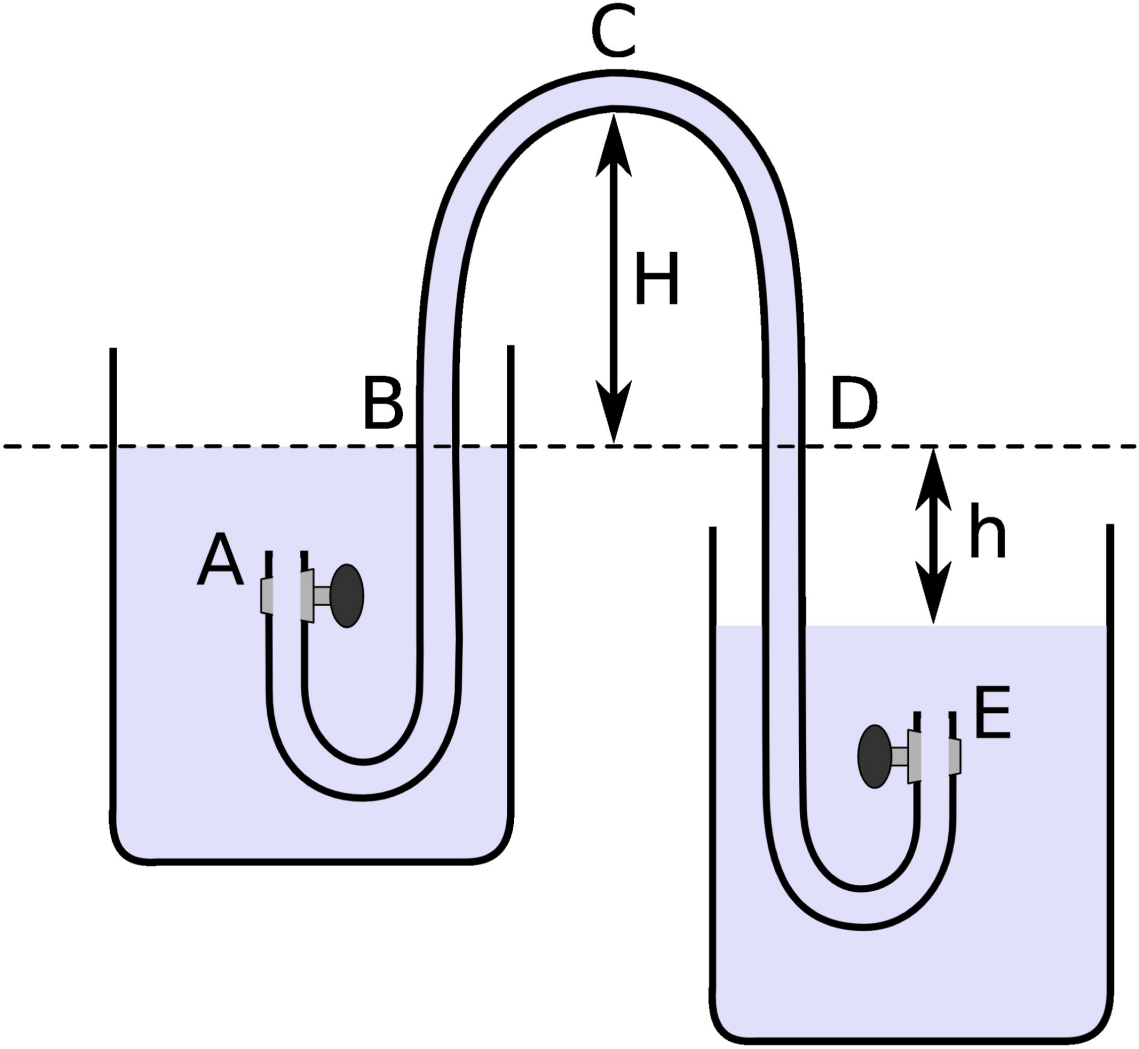
Diagram of the setup for the siphon used in our experiments.

To avoid the presence of air bubbles in our experiments, a 31 m hose lying on the ground was filled by siphoning tap water from a container. Then the hose was suspended from both ends in an almost vertical position, it was shaken and left suspended for several days to allow the remaining bubbles in the fluid to rise towards the ends of the hose. To build the siphon shown in Fig 2, we lowered both ends while maintaining them closed and pointing upwards, thus preventing the bubbles accumulated there to move towards the main section of the hose. The middle region was raised by using a frame in the form of a semicircular arc, to obtain a siphon with a height H of about 15 m between points B and C of Fig 2. This procedure formed U-shapes at both ends of the hose and allowed us to trap the bubbles in the end segments of the hose. These U-shapes where then submerged in containers filled previously with tap water and the siphon was ready for the opening of the valves at points A and E.

In our first experiments, one of the containers was raised and after opening the valves a flow of water out of both ends of the hose was observed, a clear indication of bubble formation at the top of the siphon. After many failed attempts, we succeeded in building a working siphon. To confirm the operation of a higher than 10 m siphon, we decided to exchange the heights of the containers to observe the reversal of the water flow and we added coconut milk to increase the visibility of water. However, in all the subsequent experiments a bubble was formed after opening the valves. We hypothesized that the presence of dishwasher surfactant used to clean the hose in our first successful experiment was helping to prevent air bubbles to act as nucleation centers. This idea was reinforced by the fact that surfactants are byproducts of the cellulose industry and that trees have mechanisms that prevent bubbling at negative pressures.

## Preventing the formation of bubbles

Understanding the role of gas bubbles acting as nucleation centers is the key to design successful experiments that pump water in a metastable state of absolute negative pressures, as reported by Dr. Alan Hayward in 1970. Hayward built the first mechanical pump that pulled water to heights greater than 10.33 m. Using short periods of high pressure, he decreased the size of the bubbles that could act as nucleation centers to avoid the bubbling of water in the negative pressure pulling part of the pumping cycle [10, 11].

It is widely accepted that normal boiling occurs mainly at bubbles stuck to crevices in the walls of the container and it has also been shown that bubbles trapped inside cellulose fibers play a major role as nucleation centers in carbonated beverages [10, 22, 23]. Moreover, it is known that surfactants favor the boiling of water at 100°C [24, 25]. To improve our understanding of the role played by surfactants in the prevention of bubble nucleation in a column of water at negative pressures, some simple experiments were designed to study the effects of dishwasher surfactants in the bubbling of carbonated beverages.

**Fig 3:**
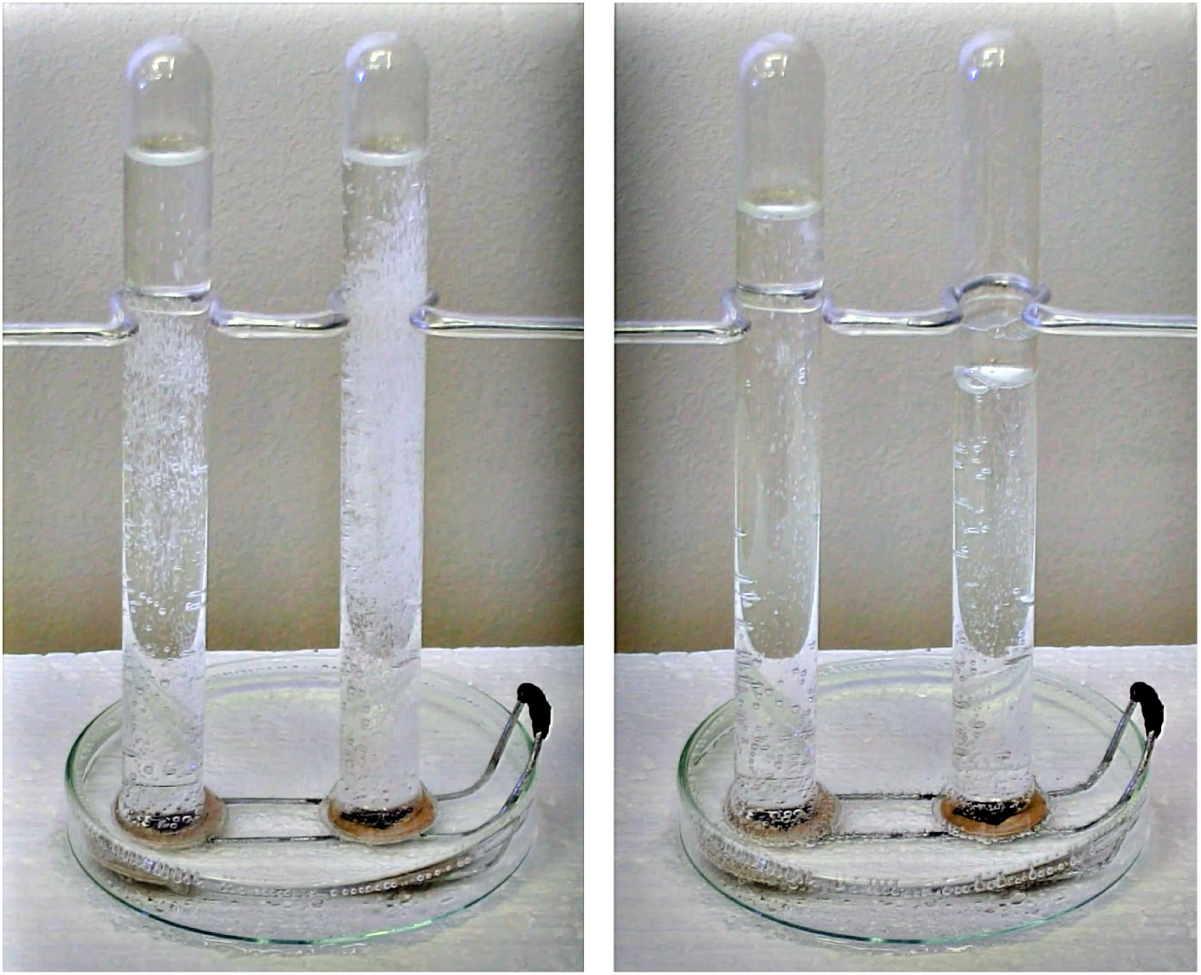
Effects of cellulose fibers (under the left test tube) and cellulose fibers wetted with surfactant (under the right test tube) on *CO*_2_ bubbling in carbonated water. Left panel: Just after the two pieces of paper were inserted below the test tubes. Right panel: 48 s later. A movie of this experiment is available as supplementary material in the online version of the paper.

Fig 3 shows a Petri dish and two inverted test tubes filled with carbonated water. A piece of paper was introduced below each test tube, increasing the bubbling of *CO*_2_ due to the presence of air bubbles trapped in the cellulose fibers of the paper which can act as nucleation centers. Under the test tube at the left a normal piece of paper was introduced, but under the tube at the right a piece of paper wetted with dishwasher surfactant was used. This simple experiment shows that cellulose fibers in both pieces of paper are acting as nucleation centers and that the surfactant changes drastically the process of bubble formation, increasing the bubbling rate and decreasing the bubble size. The left panel shows the larger rate of bubbling produced by cellulose fibers wetted with surfactant just after both pieces of paper were inserted below the test tubes. The right panel shows the larger volume of *CO*_2_ trapped at the top of the right test tube after the strong bubbling induced by the surfactant begins to decline. It is clear from this experiment that the surfactant helps degassing the fluid and its presence could explain why our siphon worked in a regime of negative pressures.

It is not completely understood how trees prevent boiling when sap is under tension, and a common assumption is that water entering through the roots by osmosis does not contain dissolved air bubbles that could act as nucleation centers. But trees can refill the xylem conduits after embolisms and although the actual mechanism is unclear [6, 7], it could be related to the mechanisms that prevent the boiling of sap when it is transported in a regime of absolute negative pressures. Only in recent years have some authors paid attention to the effects of surfactants inside the xylem of trees and its possible relationship with embolism, xylem conductivity and bubble propagation through pit membranes [26, 27]. The results of our simple experiment have not been reported before and could open new ways to understand the role played by surfactants on the transport of sap inside trees.

## Building a successful siphon taller than 10.33 meters

In our next experiments, we proceeded to use of a mix of tap water and dishwasher surfactant that turned our failed 15 m siphons into successful and robust experiments. To fill the new siphons, the mix of water and surfactant was siphoned from a container to the hose lying on the ground in an almost horizontal position. Prior to the experiment, in order to help the bubbles to rise towards the ends of the hose degassing the water, the hose was placed vertically with its ends near the upper part of a four-story building and it was shaken and left to rest for two hours.

**Fig 4:**
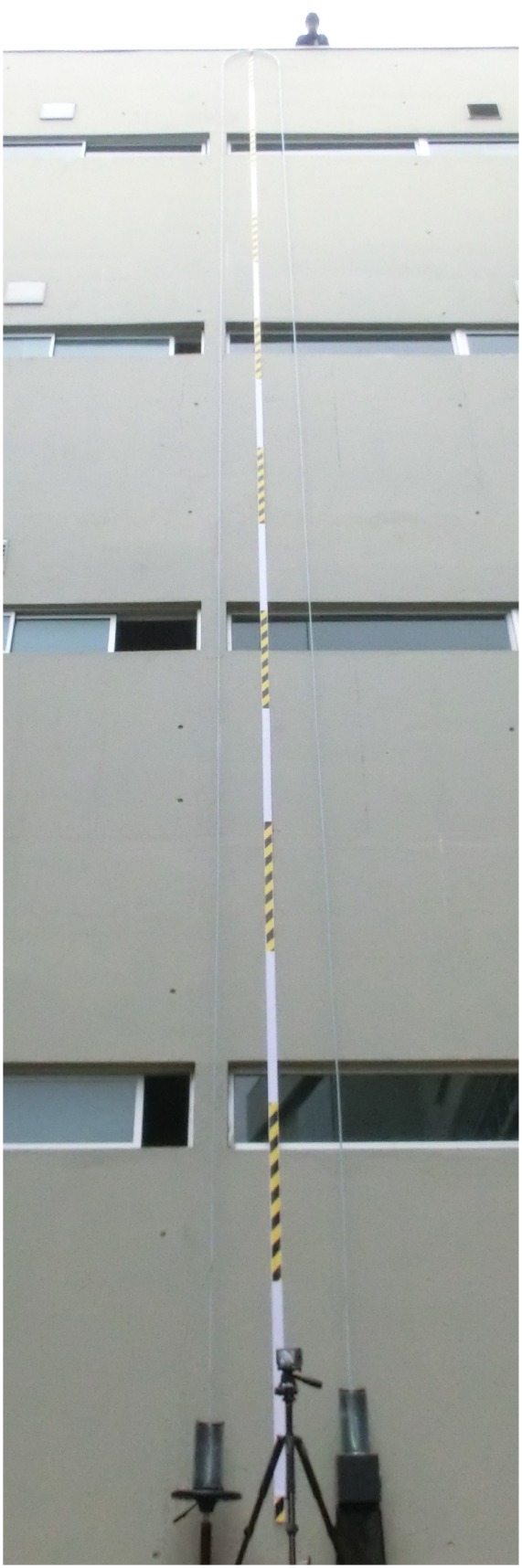
Photograph of our 15.4 m siphon. A movie of this experiment is available as supplementary material in the online version of the paper.

Fig 4 shows a photograph of the final setup before operation. This camera view captures completely the siphon setup, where the height H = 15.4 m of the middle section of the hose can be measured from this image using the marked one meter segments. For this siphon, 3 ml of concentrated dish washer surfactant was diluted in 3 L of water and 22.5 g of coconut milk was added to form a suspension in the liquid which favors the visualization of fluid flow. The density of the mix was slightly higher than that of water and its surface tension *γ* was measured using the capillary rise in calibrated glass capillaries, resulting in *γ* = 0.63 *γ*_*water*_.

**Fig 5:**
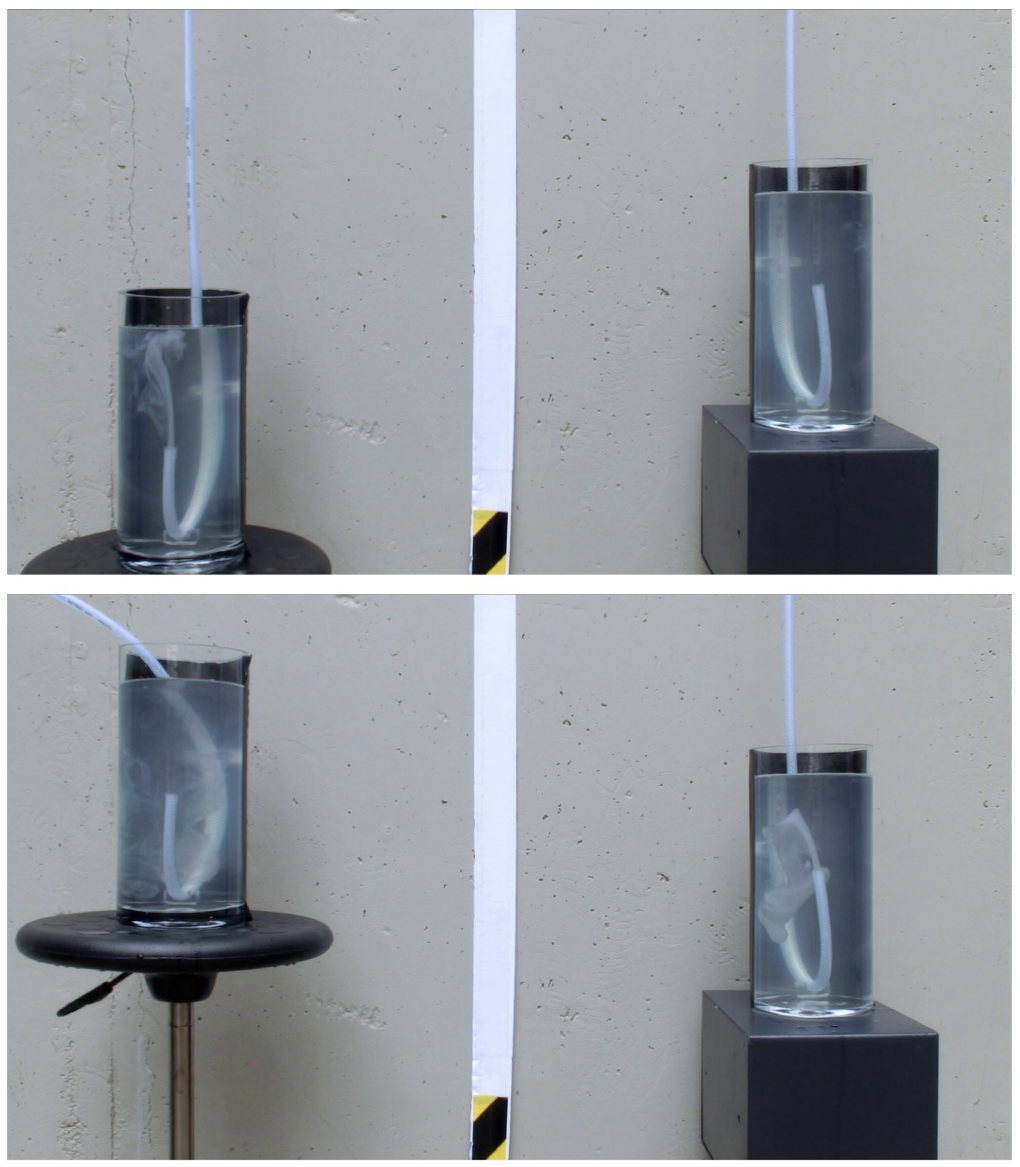
A closeup of the 15.4 m siphon during operation. The upper panel shows water being siphoned from the right container to the left container. The lower panel shows the same experiment after exchanging the heights of the containers and when water was moving from the left container towards the right container. A movie of this experiment is available as supplementary material in the online version of the paper.

Fig 5 shows a close up of this experiment (obtained with a different camera) for different heights of the left container. The upper panel shows the initial setup at t = 15 s, with the right container in a higher position and the liquid flowing from right to left. The lower panel shows the experiment at t = 102 s, after the left container was moved to a higher position and the liquid was flowing from left to right. As can be seen in the movie of this experiment, the direction of water flow was reversed after the initial flow from right to left was clearly visible. The siphon kept running for a total time of 224 s, when a bubble was formed at the upper section of the hose.

## Materials and Methods

The 15.4 m siphon was intentionally buildt with materials that can be found everywhere: a) A transparent hose with strong walls that support a pressure larger than 1 atm. b) Two stoppers to close the ends of the hose. c) A mix of tap water and dishwasher surfactant that is siphoned into the hose in order to avoid air bubbles. d) Two transparent containers partially filled with tap water. e) An arched frame and a rope that are used to rise the hose to the upper part of the building.

To visualize the water flux inside the hose we added to the mix of water and dishwasher surfactant a small quantity of coconut milk. This is optional and was used because we needed the water flux to be visible in our videos.

We used a dishwasher surfactant that is sold in our country under the brand-name ‘Quix’, which is produced by Unilever. It is a green coloured liquid, the ingredients of this particular dishwasher are not provided by the company in our country, but the composition of simmilar products comercialized in other countries made by same company contain: Anionic surfactants such as LAS (linear alkylbenzene sulfonic acid) and SLES (sodium lauryl ether sulfate), Urea, Ethanol, EDTA (Ethylenediaminetetraacetic acid), Citric Acid, Lemon Juice, Preservative, Colour and Fragrance.

Before starting a new experiment we cleaned the hose by connecting it to the tap and making the water to flow, sometimes we also added dishwasher surfactant at the initial part of the hose before connecting to the tap. Normal water from the tap was used in all of our experiments because we wanted to produce an experiment that can be easily reproduced.

## Supporting Information

S1 Video Video of *CO*_2_ bubbling. This video shows the effects of cellulose fibers (under the left test tube) and cellulose fibers wetted with surfactant (under the right test tube) on *CO*_2_ bubbling in carbonated water.
(MP4)

S2 Video Video of a 15.4 m siphon. This video shows our experiment with a 15.4 m siphon.
(MP4)

S3 Video Closeup of the 15.4 m siphon. This video shows a closeup of the previous experiment recorded with a different camera.
(MP4)
